# Medical and Physical Intelligence

**Published:** 1825-04

**Authors:** 


					341
MEDICAL AND PHYSICAL INTELLIGENCE.
THERAPEUTICS.
1. Observations on the Effect of Extract of Nux Vomica in Para-
lysis?.?Marguerite M., aged fifty-six years, chambermaid to
Madame , had suffered much from seeing this lady almost die
of a cancer of the tongue. She had attended her for nearly thirty
years. Some time previous to this, Marguerite had become affected
with a small troublesome ulcer, situated on the right side of the tongue,
exactly resembling, even as to situation, that which in her mistress had
degenerated into cancer. The mind of this woman was very highly
excited by it. The juice of herbs, frog broth (des bouillons des
grenouilles), emollient applications, soothing Tier mind, and the ex-
traction of some carious teeth, were the means which we employed, and
with success.
The small ulcer became cicatrised; nevertheless, she continued to
abandon herself to despair. She had from the first some difficulty in
assisting herself; but, as her despondency became more severe than
ever, we could perceive her, distinctly, lose by degrees the power of
motion first in the lower extremities, and then in the upper: neither did the
exhibition of bark, musk, strong jellies, nor of the preparations of steel,
nor stimulating frictions, in the least prevent or check the progress of
this paralysis. In this most miserable condition she entered the
hospital.
We consoled her in every possible manner, and cheered her with the
hope of recovery, pointing out to her the use of the extract of nux
vomica, as an infallible mean of having that hope realised. We at first
gave her but half a grain for a dose, but gradually increased it, in very
small proportions, until she took four grains night and morning; at
which time, without its being the effect of any accident, a tetanic rigidity,
accompanied with strong spasms, involuntarily and frequently repeated,
affecting the whole body, manifested themselves an hour after
taking of the medicine. She now already showed considerable signs of
relief; she could lift her legs, and support herself upon them for some
minutes. We gradually augmented the dose of the medicine, increasing
it until she took twelve, fifteen, and at last eighteen, grains during the
twenty-four hours. She, however, never took the medicine without
experiencing immediately a very sensible effect. Sometimes she had
that rigidity and general spasm, joined with slight delirium, or a species
of coma, not however alarming; all which disappeared in the space of
fourteen or fifteen minutes. She continued the nux vomica for
nearly five months, having been interrupted in its use but five or six
days, on account of a gastric irritation; which, however, a few grains
of ipecacuanha and two purgatives relieved. It procured a re-
establishment to her health, and a recovery from a severe paralysis of
both sense and motion, which had nearly proved fatal.
It is now four years since this recovery took placp, and without being
followed by any relapse.
f
S42 Medical and Physical Intelligence.
We have pushed the dose of this medicine even to the extent of
fifteen grains in a young girl, fifteen years of age, who, in consequence
of a severe fright the preceding summer, was seized with complete para-
lysis of the bladder, of the rectum, and lower extremities. Sensation
and motion of these parts were quile destroyed. After taking the me-
dicine, she experienced the very same effects as those described above;
and, when her dose was increased to eight grains, she could retain her
urine, and faeces, and move her lower extremities. She was afterwards
enabled, though but slowly, to sustain herself upon her legs, and take a
few paces, by laying hold of the bed-posts; taking at this time ten or
twelve grains of the medicine: She could walk tolerably well with as-
sistance when she left the hospital, since which time we have had frequent
opportunities of seeing her. Her legs are still weak, but she can
now walk with the help of a stick as her only support. .
We have not yet used the medicine with such decided success in any
other cases. We have always been unsuccessful in those cases which
were caused by apoplexy. In one case only, which happened in an old
man, seventy years of age, of a full habit, who was brought to the hos-
pital with a severe apoplectic attack, which we treated with copious
Wood-letting and cupping, did we succeed in restoring the sensa-
tion and motion of the right arm. The dose in this case was twenty
grains, which was continued with perseverance, and always excited
strong spasms in the whole of the diseased limb. We are, however,
convinced that, if the treatment in this case was followed with success,
it was owing to the previous copious depletion. The bad success, also,
which we have always experienced in those cases not particularly fa-
vourable, makes this conclusion appear the more correct.
These three cases have convinced us of what others had already wit-
nessed,?viz. that there are cases of palsy, which, although very severe,
may yet be cured by the extract of the nux vomica. But we ourselves
are led to conclude, in analysing the result of our experience, that they
are cases which depend upon a weakness or mobility of tl^e nervous sys-
tem, and which are not caused by any organic change followed by an
attack of apoplexy,- or any other disease which implicates either the
brain or its appendages. With respect to paralyses arising from a state
so distressing, they are generally incurable, and the extract of nux
vomica affords no relief, because it remains without acting on the cause.
{Journal General.)
SURGERY.
2. Observations on the Fracture Cradle for the lower Extremity ;
by F. Bush, Surgeon.?In the thirty-third volume of the London Me.
dicaj and Physical Journal, I published a brief description ot a machine
for the cure of fractures of the leg, with some remarks on fracture of
the thigh : the short statement is accompanied by a graphic delineation
of the " elastic iron cradle." This instrument 1 first tried on my own
person; and so completely did it answer the intention of keeping the
fractured ends of t(ie bone steadily in their proper situation, that. I was
enabled to move about with more freedom than I had ever witnessed in
Medical and Physical Intelligence . 343
any other patier.t, and actually walked about on crutches during the
whole time of the cure. Since that time (1815,) I have used this cradle
only, for all accidents of this kind. I am well aware that, were a patient
to lie in bed immovable as a log, a common pillow would supersede the
use of every contrivance of this sort; but the difficulty of keeping the
fractured ends of the bones of the lower extremities in a proper situa-
tion, in a restless patient, till union has taken place, is well known to
the surgeon, and often leads to deformity and a protracted cure.
Experience has suggested some considerable improvements in the
fracture cradle; these have been most happily carried into effect by
the ingenuity and perseverance of Mr. Alfred Green, of Sheffield,
who has succeeded in connecting the machinery for the leg with splints*
for the thigh, so constructed as to allow free motion of the knee; and
may be so altered, by the most simple process, as to admit of being
applied to a person of large or of small stature. This instrument
(which I would call " Green's elastic fracture cradle,") combines all
the advantages that can be expected from mechanism, and, as per-
formed by Mr. Green, is a valuable acquisition to the practical surgeon.
(Letter from Mr. ^ush.)
3. Method of performing the Operation of Couching among the
Hindoos.?The patient is seated on the ground opposite to the opera-
tor, the eyelids being secured by an assistant: when that is done, the
patient is directed to turn the eye upwards, and to keep it steadily in
that position; a puncture is then made, with a spear-pointed lancet,
through the sclerotica, at its lower border, about one line, or a little
more, from its junction with the cornea; and, to prevent the lancet
from penetrating too far into the substance of the eye, a piece of char-
pie or thread is wrapped round it, about three-eighths of an inch from
its point. When the puncture is completed, the lancet is withdrawn,
which is of course followed by the escape of the aqueous humour; and
a brass instrument gradually tapering to a point is introduced through
the incision, iu a direction upwards and backwards, until the point reaches
the upper margin of the lens. The lens is then depressed downwards and
backwards into the substance of the vitreous humour. After the opera-
tion, the eye is lightly covered with a piece of cotton cloth, secured by
a turn or two of a bandage. In this manner the operation is always
performed, and, if we may credit the account of the operators, with the
most wonderful success. They are unacquainted with the operation ot
extraction, and were thunderstruck at the effect ot belladonna on the
pupil.?+(Extract of a private letter from Poonah.)
PHARMACY.
4. On Pariglina, the alkaline Principle obtained from Sarsaparilla.
n the Journal de Pharmacie for November will be found an account
o this substance, as discovered by Galileo Palotta. The sarsa-
V t0 ?Ut Pleces 'n 'he usual way, and six times its weigfct
o 01 mg water poured upon it: the vessel is to be covered, to prevent
vaP^.ir *"om c?rrying toff any portion of ibe alkali. The infusion is
\o.lT? IO re,"ai" eight hours, when it i, be
2 Y
344 Medical and Physical Intelligence.
passed through a cloth: a similar quantity of water is then to be poured
on the sarsapariila, and the same process repeated as before. The two in-
fusions, when mingled together, have a deep amber colour, and are slightly
bitter and nauseous. Milk of lime is to be added to this infusion, in
such quantity as sensibly to affect turmeric paper, agitating the liquid.
The infusion now becomes brownish, and a precipitation afterwards
occurs, of a grey, pulverulent substance. This is collected on a very fine
sieve ; it is mixed, while yet humid, with water, saturated with carbonic
acid ; it is then to be dried in the sun, and reduced to tine powder. It
is next put into a matrass, with alcohol at 40?, and the ebullition kept
up for two hours. The residuum is heated with a fresh quantity of
spirit. The alcoholic liquids being mixed, they are to be distilled into
a glass receiver, as long as they are sensibly troubled. The liquid is
then to be poured out, and left undisturbed; a white powder soon sepa-
rates, which is pariglina. It is pulverulent, white, light, unchanged by
the air, of a peculiar odour, bitter, nauseous, very austere, and but
little astringent.
We liavp witnessed this operation, as performed by Mr. Garden, of Oxford-
street. Half a pound of sarsapariila, so treated, yielded but a very minute por-
tion of the pariglina, requiring the aid of a magnifying glass to satisfy us of its
real existence.?Editors.
MISCELLANEOUS.
5.?Having, in former Numbers, taken notice of the discussions
which have lately been carried on in Edinburgh, with respect to certain
new regulations relative to degrees, we think our readers will feel inte-
rested in knowing the result. The following is a copy of those which
have at length been agreed upon:?
" I. Nemo ad Doctoratus in Medicina Gradum promoveatur, prius-
quam Quadrienniuin, in hac aut alia Academia, ubi alumni ad Suinmos
in Medicina honores promoveri soliti sunt, per Sex saltern Menses
quotannis, Medicinae studio impendent; nisi
1. Qui jam evectus fuerit ad gradum Magistri Artium in Academia
quavis Scotica, vel ad parem gradum in alia Academia, opera quadrien-
nium Literis et Artibus prius dedita, quam ad Medicinae studium se
contnlerit;
2. Qui, praeter studia sua niedica in Academiis,?alio insuper anno,
? Nosocomiuin quoddain publicum, ubi aegri octoginta vel plures de-
cumbere solent, per sex saltern menses frequentaverit,?et curationi a
Medicis vel Chirurgis ibi institute adfuerit;
3. Qui per spatium unius saltern auni in re medica Exercitus vcl
Ciassis Britannicae, vel Societatis Mercaturas in India Orientali facien-
tiuin, occupatus f'uerit.
Illi autem qui quamlibet ex his conditionibus absolverit, Gramen
Doctoratus consequi liceat, postquam trienniuin in hac aut alia Acade-
mia supra detinita, Medicinse studio se dederit.
" II. Nemo Exercitationes ad Gradum Doctoratus capessendum
subeat, qui non testiinoniis idoneis ostendat,
Primo, Sequent ibus quas Scientia Medica coinplectitur Disciplinis,
sub Medicime Professoribus in hac aut alia Acadentia supra defiuita.,
semel velsaepius seoperam dedisse, scilicet,
Medical and Physical Intelligence. 845
Per curriculum Sex
Mensium;
Anatomise et Cliirurgias ....
Chemise
Materia Medicae et Pharmaceutics
Medicinae Theoretic?
Medicinae Practicae
Rei Obstetricae, et Morbis Fceminis Infantibusque
propriis
Medicinae Oinicae, scilicet curationi aegrorum in"^ Per curriculum Seit
Nosocomio Publico decumbentiuin, a Pro- f Mensium, vel per
fessoribus Medicinaj institute, et Praelecti- ? bina curricula
onibus Imbitis de iisdem aegris ... J Triuin Mensium;
duabus ex his vel pluribus, singulis annis, et etiam,
r, ( Per curriculum Tri-
? ?   \ on, Mensium;
Secundo, Duabus saltern ex sequentibus etiam Disciplinis (a seipso,
prout sibi maxime conveniant, eligendis,) in hac aut alia Academia
supra definita se operam dedisse, scilicet,
Anatomise Practicae .
Historiae Naturali .   f Per curriculum Tri-
Medicinae Legali V um saltern. Men-
? Chirurgiae Clinicaj i sium ;*
Chirurgiae Militari    J
Tertio, Praeter curriculum Medicinse Clinicae jam praescriptum,?
alio insuper anuo,?per sex saltern menses, Nosocomium quoddam
publicum hie aut alibi se frequentasse, ubi octoginta aegri decumbere
solent, et curationi a Medicis vel Cbirurgis ibi institute adfuisse.
" III. Nemo Gradum Doctoratus consequatur, qui non, unum saltern
annum, in Acadeniia Edinburgena, modo jam praescripto, Medicinae
studio impendent.
" IV. Quicunque lionores Medicinae ambierit, ante diem XXIVum
Martii, de consilio suo Facultatis Medicae Decanum certiorem faciat, et
illi tradat,
\. Autographum his verbis, c Ego , gradum Doctoratus
in Medicina ambiens, serio et sancte Medicinae Professoribus et Almae
Academiae Edinburgenae assevero, et hoc scripto meo testatum cupio,
me unum et vigesimum iEtatis Annum jam complevisse, (vel, siita res
se habuerit, ante diem proximum solennem esse completurum,) et me
fcsse liberura, scilicet nullius Chirurgi, aut Pharmacopolse, aut alius
cujusvis artificii Magistri servitioaddictum, ut Discipuluni, velTironeni,
vel Ministruni, qualis Anglice dicitur Apprentice
2. Descriptionetn Studiorum suorum, turn in Literis et Artibus, tuni
etiam in Medicina, atque de his testimonia;
3. Dissertationem Medicam Inauguralem, a seipso lingua Latina
cornpositani, et concinne conscriptam, ut Professor aliquis, a Decano
desiguandus, earn perlegat, et perlectae scriptam suani testificatiouem
apponat.
1 * Medicinae autem Clinic?, et Chirnrgiae Clinicae, uno eodemque tempore
operam dare uon.licebit.
6
346 Medical and Physical Intelligence.
" V. Ante quam tentaiuini de re medica subjiciatur Candidatus,
Facultati Medicae conventu speciali et privato ostendat, se lingua Latina
jam satis esse doctuni, quod si nou ita sit, exercitationes pro Gradu
Doctoratus, hac lingua prsecipue peragendae, non babeantur; si autem
Professoribus de hac re satisfaciat, tunc Quaestio vel viva voce vel
scripto, statim habenda est, de variis quas Scientia medica complectitur
disciplinis; eo ut nemini, nisi Scientia turn Literaruru humaniorum,
turn Medicinae probe imbuto, Candidatorum numero retineri liceat.
" VI. Si quis, hac quasstione privatim babita, Candidatorum numero
retineri dignus non existimabitur, duabus saltern ex supradictis (Sect.
II.) disciplinis, sub Medicinae Professoribus in hac aut alia Academia
supra deiinita, iterum operam det, antequam exercitationes iterum
subeat.
" VII. Si Candidatus,. qu.aest.ione privatim habita, proraoveri merebi-
tur,. illi statim tradantur duae Morborum Historiae, cum quapstionibus
subjunctis, ut, commentis lingua Latina scriptis, illas illustret, his
commoda responsa reddat; turn Historias ita illustratas, una cum
Responsis suis, post decern dies Professoribus proponentibns tradat,
atqueeadem, die Vlto Julii, coram Facuhate Medica defendat.
" VIII. Candidato, si, post primum- periculnm factum, probatus
fuerit, Dissertationem suam inauguralem prelo subjicere liceat, nec ta-
men imperetur: Dissertations cujusque excusae quadraginta exemplaria,
Facultatis Medicae Decano, die Vlto Julii, tradantur.
" IX. Si Candidatus iterum a Medicinae Facilitate fuerit probatus,
ejusdem Facultatis Decanus quae gesta fuerint Seuatui Academico re-
nunciabit; cujus approbatione et auctoritate Candidatus Dissertationem
suam in Comitiis Academicis, die Xlmo Julii, lingua Latina, defendere,
ct, si excusa fuerit, edere jubeatur: Turn, si Senatui placuerit, laboris
et studiorum proemium, summos in Medicina Honores, Graduiu nenipe
Doctoratus, more solenni, Xllmo Julii, consequatur.
" X. Facultas Medica, quo major sit horum omnium solennitas,
semper intra Academiae Pomoeria, hora decima ante meridiem, diebus
supradictis, conveniet. Et si quis Candidatus, sine gravi causa, bora
abfuerit statuta, occasione neglecta, huic, nec ad ulteriora pericula pro-
gredi, nec Gradum Doctoratus hac vice assequi, licebit.
" N.B.?Hiec sunt Statuta de studiis, pro Gradu Doctorali, omnium
qui posthac ad Medicinae studium se conferant. Eorum autem qui
annis 1825, 1826, aut 1827, honores in Medicina ambiant, studia et
exercitationes, Statutis Solennibus jam (anno scilicet 1823) editis, diri-
yenda sunt."
6. Medical Society of London.?In conformity with the will of the
Jate Dr. Anthony Fothergill, the Society otfers the annual gold
medal, value twenty guineas, for the best Dissertation on a subject pro-
posed by them; for which prize, the learned of all countries are invited
as candidates.
The subject for this year is " The Nature and Treatment of Carci-
noma."
1. Each Dissertation must be delivered to the Registrar in the Latiu
or English language, on or before the 1st of December.
Medical and Physical Intelligence, 347
2. Witb it must be delivered a sealed packet, with some motto or
device on the outside, and within the author's name and designation;
and the same motto or device must be put on the Dissertation, that the
Society may know how to address the successful candidate.
S. No paper in the hand-writing of the author, or with his name
affixed, can be received; and if the author of any paper shall, directly
or indirectly, discover himself to the Committee of Papers, or to any
member thereof, such paper will be excluded from all competition for
the medal.
4. The prize-medal will be presented to the successful candidate, or
his substitute, at the anniversary meeting of the Society, in March, 1826.
. 5. All the Dissertations, the successful one excepted, will be returned
(if.desired), with the sealed packet unopened.
* * The subject of the Dissertation for tJiQ year 1826-7, is " Conta-
eion and Infection.'
7. Vaccine Establishment.?Copy of Report received by the Secre-
tary of State for the Home Department, from the Board of the
Vaccine Establishment; 12th February, 1825.
To the Right Honourable Robert Peel, Secretary of State for
the Home Department, &c. &c. &c.
Sir,?The importance and value of this Establishment is'made ma-
nifest daily, by the immediate and certain return it makes to all appli-
cations for means of protection against one of the greatest scourges, in
the form of disease^ which has ever afflicted mankiud.
It belongs to the improvidence of the lowest orders of the people to
L.main careless and inactive, so long as the hour of danger is distant
and uncertain, and to become, perhaps, unreasonably alarmed whenitar-
rives. Our calls, therefore, are generally accompanied by information that
the small-pox is committing great ravages in the neighbourhood, and
the demands for assistance are urgent and pressing. These remarks
apply to our own country more particularly; but we have frequent ap-
plications also from abroad, and even from vaccine institutions esta-
blished in foreign capitals, where it is admitted to be difficult to keep
up Ihe supply ; and the request for matter is made under a conviction
that that which is received from the Establishment is more genuine and
efficacious. Happily the arrangements which we have made enables us
to supply every want, whenever and from whatever quarter it comes to
us; and we have sent out 77?800 charges of lymph since our last
Report.
It cannot be necessary now to enter upon an estimate of the compa-
rative merits of vaccination and inoculation, as protectives against the
small-pox; but the Board has been engaged in endeavouring to ascer-
tain what proportion of persons vaccinated take the small-pox after,
wards. By the information which we obtain from our stationary
vaccinators in the metropolis, it seems that not more than eighteen out
the 8,000 which are vaccinated, upon an average, annually, are suscep-
tible of the variolous disease afterwards. The returns from the
corresponding vaccinators in the country are less favourable; but we
are fully justified in concluding, that the number of those who.take the
348 Medical and Physical Intelligence.
small.pox after vaccination, and pass through a safe and harmless dis-
ease, is not greater than the number of those who die under inoculation.
The estimate of this loss, where inoculation was greatly practised, was
one in 300.
By the Bills of Mortality, we find that 725 persons have died of the
small-pox this year, within the range of these Bills; but we have not
been made acquainted with any fatal case after vaccination.
We are, &c.
(Signed) Henry Halford,
i President of the Royal College of Physicians.
William Norris,
! President of the Royal College of Surgeons.
Wm. Lynn, Vice-President,
Wm. G. Maton, m.d. }
Hy. James Cholmrley, m.d. {Censors of the Royal
Francis Willis, m.d. [Coll, of Physicians.
John Warburton, m.d. j
? Clemt, Hue, m.d. Registrar.
8. Tabic of Admissions into the Small-Pox Hospital, during the
<year 1824.?
Class.
Characters of the Disease.
Small-pox in the unprotected, in its
greatest virulence ???*..
(Confluent malignant.)
?? , in its
second degree"of virulence
(Simple confluent.)
third degree of virnlence
( Coherent.)
hi its
in its
mild form
( Distinct.)
Total in the unprotected
Small-pox of the confident, coherent,
and distinct kinds, occurring subse-
quent to Vaccination
(Modified.)
Small-pox occurring subsequent to Ino-
culation
C Secondary.)
Eruptive diseases, not Small-pox > ?
Total?
Admissions,
12
46
55
35
148
45
199
Deaths.
12
*7
14
54
54
Table of the Ages of the several Cases admitted in 1824.
Under seven years of age* ? ?
Between seven and fourteen
Adults
Total* ??????????
33
15
151
199
19
2
33
54
Total vaccinated in the year 1824-
34$
? meteorological journal,
From February 20 to March 19, 1825.
By Messrs. William Harris and Co. 50, liolborn, London*
Rain
gauge
Feb.
20
21
22
23
24
25
26
27
28
Mar
A
2
3
4
5
6
7
8
9
10
11
12
13
14
15
16
17
18
19
.05
.03
BARON.
30.17
30.27
30.31
30.07
30.10
30.22
30.13
29.63
29.45
30.26
30.33
30.21
30.03
30.20
30.22
30.00
29.48
29.54
29.58
29.30
29.30
29.48
30.06
29.96
29.56
30.07
30.13
30.10
30.00
30.00
30.00
29.95
30.00
30.10
30.28
30.41
30.44
29.19
29.06
29.35
29.83
30.14
29.98
29.71
30.16
30.11
30.12
29.97
30.06
29.98
29.95
30.10
30.20
30.33
30.44
SO. 44
DE
LUC's
HYG.
WIND.
9 a.m.
W
W
N
SE
SSE
NE
ENE
ssw
NW
sw
w
wsw
w
NW
w
s
SvV
w
sw
NW
SW
E
E
NE
S
SSE
E
10P.M.
w
N
SE
ESE
E
ENE
S
NW
SW
SE
SW
ssw
NNE
NW
S
SSW
sw
sw
sw
WNW
NNE
NE
E
ENE
ENE
S
SE
E
ATMOSPHERIC
VARIATIONS.
9 A.M.
Cloud.
Foggy
Cloud.
Rain
Fine
Rain
Fine
Cloud.
Rain
SI. Fog
Rain
Cloud
Rain
Fine
Rain
Cloud.
Sleet
Fine
2 P.M.
lOp.M.
Cloud.
Fine
Snow
Raiu
Fine
Cloud.
Fine
Cloud,
Fine
Cloud
Sleet
Cloud
Rain
Cloud,
Hail
Fiue
Cloud.
Fine
Cloud.
Rain
Cloud.
Fine
Cloud.
Fine
Cloud.
Fine
The quantity of rain fallen in the month of February,
was 60.100ths of an inch.

				

